# Mauriac Syndrome: Growth and Clinical Outcomes After 2.5 Years of Automated Insulin Delivery Treatment

**DOI:** 10.1210/jcemcr/luaf282

**Published:** 2026-01-19

**Authors:** Hanine Alarab, Lina Merjaneh, Kelsey B Eitel

**Affiliations:** Department of Pediatrics, University of Washington, Seattle, WA 98145, USA; Pediatric Endocrinology and Metabolism, Texas Children's Hospital, Baylor College of Medicine, Houston, TX 77030, USA; Department of Pediatrics, University of Washington, Seattle, WA 98145, USA

**Keywords:** Mauriac syndrome, hepatic glycogenosis, type 1 diabetes mellitus, automated insulin delivery, insulin pump

## Abstract

Mauriac syndrome is a rare complication of type 1 diabetes mellitus (T1D) with chronically elevated hemoglobin A1C (HbA1c) that is characterized by short stature, delayed puberty, cushingoid features, and hepatic glycogenosis. We report a 14-year-old male patient with T1D managed with multiple daily insulin injections who presented with growth failure and delayed puberty in the setting of several years of HbA1c > 12% (SI: > 108 mmol/mol) (reference range, < 5.7% [SI: < 39 mmol/mol]). He was initially suspected to have growth hormone deficiency, failed a growth hormone stimulation test and received growth hormone treatment without an increase in height velocity. After several months, he presented with abdominal distention due to new hepatomegaly. Laboratory evaluation revealed transaminitis with normal synthetic function and absence of cholestasis. Liver biopsy confirmed hepatic glycogenosis. Treatment included T1D management re-education, psychosocial support, and transition to automated insulin delivery (AID). AID resulted in decreased HbA1c level, normalized liver enzymes, resolution of hepatomegaly, puberty progression, and increased linear growth in line with his mid-parental height. This patient demonstrated that growth failure and delayed puberty can precede hepatic glycogenosis and that AID is a safe and effective treatment option for patients with Mauriac syndrome.

## Introduction

Mauriac syndrome (MS) is a rare complication of type 1 diabetes mellitus (T1D) in children and adolescents with a persistently elevated hemoglobin A1C (HbA1c) level [1]. The cardinal features include growth failure, delayed puberty, hepatic glycogenosis, and dyslipidemia [1-3]. The pathophysiology of growth impairment in MS may include insufficient glucose uptake for adequate tissue energy, decreased circulating insulin-like growth factor 1 (IGF-1), growth hormone resistance, and hyperadrenocorticism [3]. Other features can include cushingoid body habitus, proximal muscle wasting, retinopathy, nephropathy, neutropenia, and persistent lactic acidosis [3, 4].

The exact incidence remains unknown due to limited reported cases. Recent literature includes approximately 25 documented cases in the past decade [3, 4]. Youth and adolescents with barriers to care are at high risk of persistently elevated HbA1c level, placing them at higher risk of MS diagnosis and may lead to challenges in treating MS [5]. Barriers may include health literacy, parental education, parental and school supervision, mental health comorbidities, diabetes distress, and socioeconomic stressors [5].

Treatment of children and adolescents with MS includes intensification of insulin regimen to improve glycemic control, which in turn leads to an increase in linear growth, progression of endogenous puberty, and resolution of hepatic glycogenosis [1-3]. A risk of intensified insulin regimen in MS is worsening or rapid development of retinopathy or nephropathy [3]. Automated insulin delivery (AID) is associated with more time in range, less frequent hypoglycemia, decreased diabetes distress, reduced fear of hypoglycemia, improved sleep quality, and improved quality of life [6, 7]. Given the tighter glycemic control and improved psychosocial functioning with AID, it could be an ideal treatment selection for patients with MS. We present a patient with MS who was successfully treated with AID with improvement in long-term growth outcomes and no complications.

## Case Presentation

A 14-year-old male patient with T1D, managed on multiple daily insulin injections (total daily dose 1 unit/kg/day) and continuous glucose monitoring, presented with concerns of short stature. T1D was diagnosed at age 10 years with 4 positive pancreatic islet autoantibodies. He had persistently elevated HbA1c levels ranging 12.7% to >14% (SI: ranging 115 mmol/mol to >130 mmol/mol) (reference range, < 5.7% [SI: < 39 mmol/mol]) and 2 episodes of diabetic ketoacidosis in the 2 years preceding presentation for the evaluation short stature.

**Figure 1.**
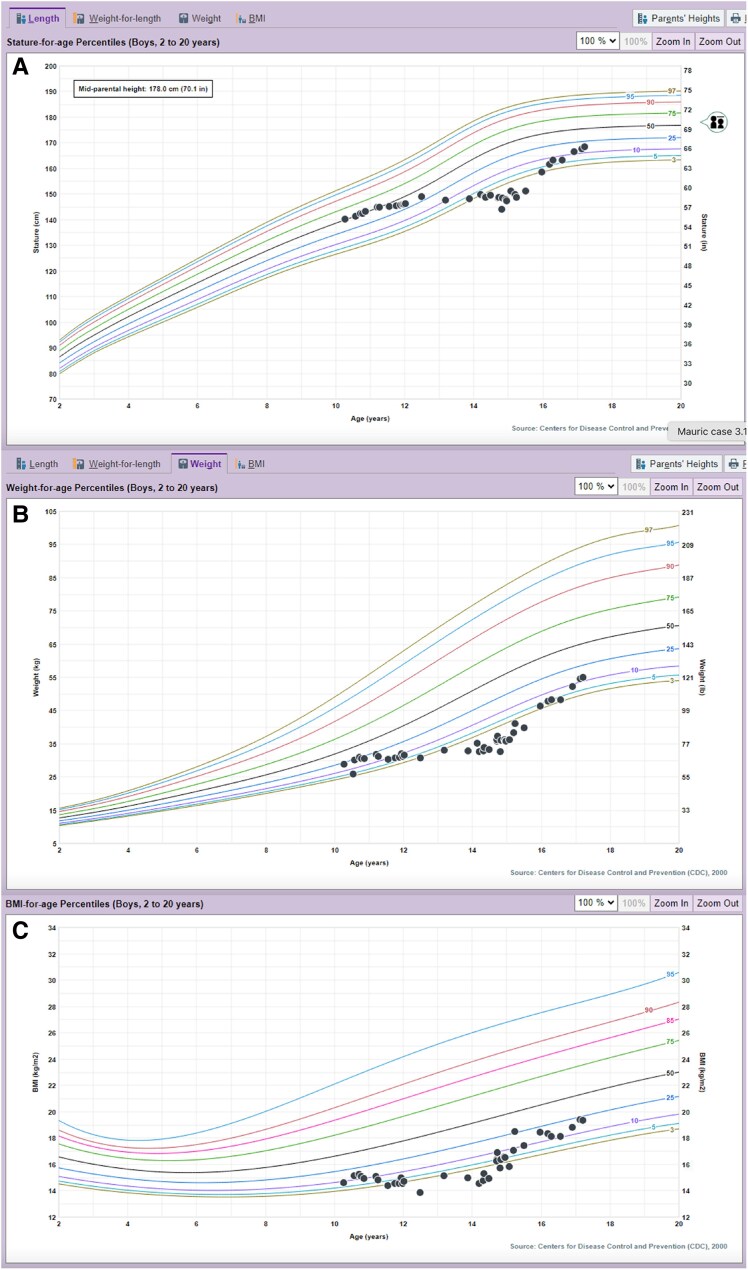
Centers for disease control and prevention (CDC) growth charts for length (A), weight (B), and body mass index (C) boys 2-20 years.

He had moderately severe depression symptoms (Patient Health Questionnaire-9 [PHQ-9] score 17, PHQ-9 score ranges 0 to 27, normal less than 4) and high diabetes distress (Problem Areas in Diabetes-Teen [PAID-T]) score 54; PAID-T score ranges 14-84, normal less than 40). From diagnosis to 14 years old, his height declined from the 50th to the 3rd percentile with height velocity 1 cm/year and weight declined from the 25th to the 0.5%ile with body mass index Z-score −2.89 (Fig. 1). His mid-parental height is 70.1 inches (50th percentile). Abdominal examination was normal with no organomegaly. He was Tanner 2, with testes measuring 6 cc bilaterally. Laboratory values are shown in Table 1 with IGF-1 93 ng/mL (SI: 12.2 nmol/L) (reference range, 14y: 148-551 ng/mL; [SI: 19.4-72.3 nmol/L]). Bone age was delayed at 11 years.

**Table 1.** Laboratory evaluations

Laboratory test	Short stature evaluation (14y, 0m)	Emergency room evaluation (14y, 8m)	Follow-up after 1 year on AID (15y, 11m)	Conventional (reference range)	SI units (reference range)
Follicle stimulating hormone	<0.7 mIU/mL (SI <0.7 IU/L)	—	1.9 mIU/mL (SI 1.9 IU/L)	0-10 mIU/mL	0-10 IU/L
Luteinizing hormone ultra-sensitive	<0.02 mIU/mL (SI <0.02 IU/L)	—	—	Tanner 2: 0.5-1.76 mIU/mL	0.5-1.76 IU/L
Luteinizing hormone	—	—	1.1 mIU/mL (SI 1.1 IU/L)	12-17y: < 4.8 mIU/mL	< 4.8 IU/L
Total testosterone by liquid chromatography-mass spectrometry	<7.0 ng/dL (SI <0.24 nmol/L)	—	215 ng/dL (SI 7.46 nmol/L)	Tanner 2: 8-66 ng/dL	0.28-2.29 nmol/L
Insulin-like growth factor 1	93 ng/mL (SI 12.2 nmol/L)	—	405 ng/mL (SI: 53.3 nmol/L)	14y: 148-551 ng/mL; Tanner 2: 106-432 ng/mL	19.4-72.3 nmol/L
Insulin-like growth factor binding protein-3	3 µg/mL (SI 3 mg/L)	—	4.9 µg/mL (SI 4.9 mg/L)	14y: 2.6-6.3 µg/mL; Tanner 2: 3.5-5.7 µg/mL	2.6-6.3 mg/L
Thyroid stimulating hormone	2.13 µIU/mL (SI 2.13 mIU/L)	—	—	0.6-5.6 µIU/mL	0.6-5.6 mIU/L
Free thyroxine	1.2 ng/dL (SI 15.5 pmol/L)	—	—	0.8-2.0 ng/dL	10.3-25.8 pmol/L
Tissue transglutaminase	<1 U/mL (SI <1 kU/L)	—	—	<14 U/mL	<14 kU/L
Immunoglobulin A level	110 mg/dL (SI 1.1 g/L)	—	—	35-252 mg/dL	0.35-2.52 g/L
Aspartate aminotransferase	36 IU/L (SI 36 U/L)	167 IU/L (SI 167 U/L)	19 IU/L (SI 19 U/L)	5-41 IU/L	5-41 U/L
Alanine aminotransferase	56 IU/L (SI 56 U/L)	153 IU/L (SI 153 U/L)	18 IU/L (SI 18 U/L)	5-52 IU/L	5-52 U/L
Alkaline phosphatase	133 IU/L (SI 133 U/L)	120 IU/L (SI 120 U/L)	345 IU/L (SI 345 U/L)	124-474 IU/L	124-474 U/L
Gamma-glutamyl transferase	68 IU/L (SI 68 U/L)	83 IU/L (SI 83 U/L)	16 IU/L (SI 16 U/L)	15-85 IU/L	15-85 IU/L
Total bilirubin	—	0.2 mg/dL (SI 3.4 µmol/L)	—	0.1-1.2 mg/dL	1.7-20.5 µmol/L
Direct bilirubin	—	0 mg/dL (SI 0 µmol/L)	—	0-0.3 mg/dL	0-5.1 µmol/L
Prothrombin time	—	11.5 seconds (SI 11.5 seconds)	—	12.5-15.2 seconds	12.5-15.2 seconds
Activated partial thromboplastin time	—	20 seconds (SI 20 seconds)	—	25-35 seconds	25-35 seconds
Lipase	—	85 IU/L (SI 85 IU/L)	—	25-120 IU/L	25-120 IU/L
Lactate dehydrogenase	—	244 IU/L (SI 244 IU/L)	—	125-253 IU/L	125-253 IU/L
Albumin	—	3.6 g/dL (SI 36 g/L)	—	3.5-5.0 g/dL	35-50 g/L
Glucose	—	257 mg/dL (SI 14.3 mmol/L)	—	70-99 mg/dL	3.9-5.5 mmol/L
HbA1c	13.2% (SI 121 mmol/mol)	>14% (SI > 130 mmol/mol)	10.8% (SI 95 mmol/mol)	<5.7%	< 39 mmol/mol
Total cholesterol	214 mg/dL (SI 5.54 mmol/L)	204 mg/dL (SI 5.28 mmol/L)	—	125-200 mg/dL	3.24-5.17 mmol/L
Low-density lipoprotein	167 mg/dL (SI 4.32 mmol/L)	152 mg/dL (SI 3.93 mmol/L)	—	63-130 mg/dL	1.63-3.36 mmol/L
High-density lipoprotein	51 mg/dL (SI 1.32 mmol/L)	62 mg/dL (SI 1.60 mmol/L)	—	38-75 mg/dL	0.98-1.94 mmol/L
Triglycerides	305 mg/dL (SI 3.45 mmol/L)	476 mg/dL (SI 5.38 mmol/L)	—	60-135 mg/dL	0.68-1.53 mmol/L
Lactate	—	63.9 mg/dL (SI 7.1 mmol/L)	—	4.5 to 19.8 mg/dL	0.5-2.2 mmol/L
White blood cells	—	3.5 K/µL (SI 3.5 × 10⁹/L)	—	4.5-11 K/µL	4.5-11 × 10⁹/L
Hemoglobin	—	11.6 g/dL (SI 116 g/L)	—	13-16 g/dL	130-160 g/L
Platelets	—	183 K/mm³ (SI 183 × 10⁹/L)	—	140-400 K/mm³	140-400 × 10⁹/L
Absolute neutrophil count	—	1015/mm³ (SI 1.015 × 10⁹/L)	—	1500-7000/mm³	1.5-7 × 10⁹/L

Abbreviation: AID, automated insulin delivery.

After excluding other causes of poor growth, including hypothyroidism, celiac disease, and adrenal insufficiency, the patient underwent growth hormone stimulation testing using clonidine and arginine after priming with estrogen (2 mg nightly for 2 consecutive nights preceding stimulation test), which showed subnormal peak growth hormone levels of 1.2, 4.5, 4.4, 1.6, 0.7, 2.2, 4.6, 3.4 ng/mL (SI: 1.2, 4.5, 4.4, 1.6, 0.7, 2.2, 4.6, 3.4 μg/L) (reference range, > 6 ng/mL [SI: > 6 μg/L]). Magnetic resonance imaging of the brain showed a normal pituitary gland. Growth hormone therapy was initiated at a dose of 0.24 mg/kg/week. He received growth hormone for 5 months without height change. IGF-1 levels were not re-evaluated after starting treatment, as the patient did not present to his regular follow-up visit.

At age 14 years and 8 months, the patient presented to the emergency department with progressive abdominal distention and discomfort.

## Diagnostic Assessment

Physical examination revealed diffuse abdominal distension, no ascites or fluid wave, hepatomegaly 5.5 cm below right costal margin, and splenomegaly 2 cm below left costal margin. Laboratory values are shown in Table 1. Computed tomography scan of his abdomen demonstrated an enlarged liver measuring 18 cm in craniocaudal dimension with homogeneous enhancement, mild splenomegaly, and no discrete masses.

Further workup for infectious and autoimmune etiologies was negative for Epstein–Barr virus, cytomegalovirus, adenovirus, hepatitis A, B, and C, anti-smooth muscle, anti-nuclear antibodies, and liver kidney microsomal antibodies. He subsequently underwent a diagnostic liver biopsy. Pathology revealed diffusely enlarged hepatocytes with sharp polygonal edges and staining consistent with increased glycogen stores, glycogenated nuclei (a histopathological sign of excessive glycogen accumulation) present in ∼20% of hepatocytes and no evidence of fibrosis, steatosis, or cholestasis.

Genetic testing on blood was done with Trio whole exome sequencing, which revealed 2 heterozygous variants of uncertain significance in the glycogen phosphorylase L (*PYGL*) gene (c.1900G>C and c.1757C>T). Glycogen phosphorylase activity was mildly reduced to 5.05 umol/min/g tissue (SI: 8.45 × 10^−5^ mol/s/kg tissue) (reference range, 7.4-13.2 umol/min/g tissue [SI: 1.23 to 2.2 × 10^−4^ mol/s/kg tissue]), consistent with glycogen storage disease VI (GSDVI) carrier status. Alpha-1 antitrypsin (*A1AT*) phenotype testing was consistent with heterozygous MZ phenotype (one normal allele M and one significantly deficient allele Z) and quantitative testing confirmed mildly reduced A1AT protein levels of 52 mg/dL (normal 100-190 mg/dL), which can increase the risk of hepatic injury.

## Treatment

The patient reported compliance with growth hormone therapy, as short stature was his primary concern. However, due to the lack of height increase and the diagnosis of Mauriac syndrome, growth hormone therapy was discontinued. The family received T1D management re-education with a multidisciplinary diabetes team and increased psychosocial support through regular social work appointments. The patient endorsed burnout, difficulty with motivation, and parental conflict with perceived blame for elevated blood glucose levels. His insulin regimen was intensified initially with multiple daily injections followed by initiation of AID with Tandem t:slim X2™ control IQ system 5 months later.

## Outcome and Follow-Up

Follow-up after 1 year on AID was notable for HbA1c 10.8% (SI: 95 mmol/mol), time in range on continuous glucose monitoring 21%, resolution of hepatomegaly and transaminitis (Table 1), increased height velocity of 15 cm/year, improved height (3rd percentile, Fig. 1), and progression of endogenous puberty on examination and laboratory assessment (Table 1). His bone age continued to be delayed (bone age 11 years 6 months, chronological age 15 years 11 months) with predicted adult height of 188 cm (74 inches).

At 2.5 years from initial MS diagnosis (2 years from starting AID), his height was at the 16th percentile (Fig. 1) with HbA1c 8.9% (SI: 74 mmol/mol). He had no microalbuminuria and no evidence of retinopathy on the diabetes eye exam. He had a low level of diabetes distress (PAID-T score 29) and minimal depression symptoms (PHQ-9 score 2). He attended a summer camp for adolescents with diabetes, was excited to meet peers with diabetes and became more engaged with diabetes management.

## Discussion

This case demonstrates that patients with MS may initially present with short stature and delayed or stalled puberty prior to hepatic involvement. A high index of suspicion for MS is needed in patients with T1D, persistently elevated HbA1c level, and growth failure.

The pathophysiology of hepatic glycogenosis is not fully understood but is likely multifactorial. It is driven by hyperglycemia, intermittent supraphysiologic insulin exposure, and enzymatic imbalances in glycogen metabolism [3], creating a cycle of excessive glycogen storage with inadequate degradation, leading to progressive hepatomegaly [4]. A genetic cause of MS was demonstrated in a liver glycogen phosphorylase kinase mutation, which inhibits glycogenolysis via glycogen phosphorylase during periods of hyperglycemia [8].

Our patient was a heterozygous carrier of *A1AT* gene, which is associated with mildly low A1AT levels. This MZ phenotype is not associated with progressive fibrosis in isolation but can contribute to hepatic injury with concomitant processes [9]. The patient's *PYGL* variants present a unique potential implication on glycogenolysis. *PYGL* encodes liver glycogen phosphorylase, the rate-limiting enzyme in glycogenolysis, and 2 pathogenic variants result in glycogen storage disease type VI (GSDVI) [10]. GSDVI can cause hepatomegaly, hypoglycemia, and poor growth; however, this would not be reversible with improved glycemic control. Our patient had mildly reduced liver glycogen phosphorylase levels, consistent with GSDVI carrier status. It is possible the carrier status may predispose to hepatic glycogenosis during periods of hyperglycemia. Future studies should evaluate the role of *PYGL* in people with T1D and hyperglycemia.

MS treatment is focused on lowering HbA1c levels with insulin regimen intensification, which results in increase in linear growth, progression of puberty, and resolution of hepatic glycogenosis [3, 4]. Catch-up growth may be limited in some cases [3]. One case report discussed standard insulin pump therapy with manual dose intensification and noted no retinopathy after 2.5 years, but growth data were lacking [11]. There is one prior case report on AID use for MS treatment, in which there was minimal increase in linear growth and no reported long-term clinical outcomes [12]. Our case adds valuable insight into the long-term outcomes using AID in MS and demonstrates robust increased height velocity, rapid catch-up growth, and progression of puberty. Based on his current height and bone age, he is expected to achieve a near adult height in line with his genetic potential.

This case reports on retinopathy outcomes after AID initiation in a patient with MS. Our patient did not develop retinopathy, but caution should be taken with rapidly lowering glucose given the increased risk of rapid progression of retinal disease [3, 13]. The underlying mechanism is thought that rapid glucose reduction in diabetes can worsen retinal damage by disrupting the retina's adaptation to chronic hyperglycemia, leading to worsened hypoxia due to impaired blood flow regulation [13]. This may trigger an acute imbalance of growth factors like vascular endothelial growth factor and IGF-1, accelerating neovascularization and diabetic retinopathy progression [13]. Additionally, increased oxidative stress and hemodynamic changes further contribute to endothelial dysfunction and microvascular damage, exacerbating retinal injury [13]. AID achieves higher time in range than multiple daily injections and standard insulin pumps [6] and therefore may pose a higher risk of retinopathy. Some AID systems can be programmed to have a higher glucose target, lower basal rate, higher correction factor, or higher active insulin time in order to achieve improved glycemic control in a gradual, stepwise fashion. AID system selection may be influenced by the ability to program these doses and variables.

In addition to an intensified insulin regimen, increased mental health, social, and economic support is important for improved glycemic outcomes [5]. This may include therapy, fostering family support, encouraging nonjudgemental communication with family members, engaging in a community of peers with diabetes such as diabetes summer camp, and financial assistance. AID is likely to have psychosocial benefits in patients with MS as it has been associated with decreased diabetes distress, reduced fear of hypoglycemia, improved sleep quality and improved quality of life [7]. Our patient had improvements in diabetes distress, depressive symptoms, and noted positive effects from social support at diabetes camp.

Mauriac syndrome persists as a rare but severe complication of T1D and may initially present with growth failure and delayed or stalled puberty, prior to liver disease. AID is associated with improved glycemic control and psychosocial functioning and therefore may improve clinical outcomes in several different ways.

## Learning Points

- Mauriac syndrome persists as a rare but severe complication of T1D with elevated HbA1c level and may initially present with growth failure and delayed or stalled puberty prior to hepatic glycogenosis.
- The *PYGL* gene encodes liver glycogen phosphorylase, the rate-limiting enzyme in glycogenolysis, and our patient's variant was consistent with GSDVI carrier status, which may predispose to hepatic glycogenosis during periods of hyperglycemia.
- Automated insulin delivery is associated with improved glycemic control and psychosocial functioning and can be a safe and effective treatment option for Mauriac syndrome.
- Mental health, social, and economic support are crucial to improve glycemic outcomes and growth potential in Mauriac syndrome.

## Contributors

All authors made individual contributions to authorship. H.A., L.M., and K.E. were involved in the diagnosis and management of this patient and manuscript generation and submission. All authors reviewed and approved the final draft.

## Data Availability

Data sharing is not applicable to this article as no datasets were generated or analyzed during the current study.

## Abbreviations

AID: automated insulin delivery
GSDVI: glycogen storage disease VI
HbA1c: glycated hemoglobin
IGF-1: insulin-like growth factor 1
MS: Mauriac syndrome
PAID-T: Problem Areas in Diabetes-Teen
PHQ-9: Patient Health Questionnaire 9
T1D: type 1 diabetes
